# Biocompatibility of a HA/β-TCP/C Scaffold as a Pulp-Capping Agent for Vital Pulp Treatment: An In Vivo Study in Rat Molars

**DOI:** 10.3390/ijerph18083936

**Published:** 2021-04-08

**Authors:** Julia Guerrero-Gironés, Antonia Alcaina-Lorente, Clara Ortiz-Ruiz, Eduardo Ortiz-Ruiz, María P. Pecci-Lloret, Antonio José Ortiz-Ruiz, Francisco Javier Rodríguez-Lozano, Miguel R. Pecci-Lloret

**Affiliations:** 1 Special Care and Gerodontology Unit, IMIB-Arrixaca, Campus Regional de Excelencia Internacional “Campus Mare Nostrum”, University of Murcia, 30008 Murcia, Spain; julia.guerrero@um.es (J.G.-G.); fcojavier@um.es (F.J.R.-L.); miguelramon.pecci@um.es (M.R.P.-L.); 2 Department of Integral Pediatric Dentistry, University of Murcia, 30008 Murcia, Spain; alcainalorentea@gmail.com (A.A.-L.); ajortiz@um.es (A.J.O.-R.); 3 Department of Physiology, School of Medicine and Biosanitary Research Murcian Institute (IMIB), University of Murcia, 30120 Murcia, Spain; clara@um.es; 4 Department of Histopathology, University Hospital Virgen de la Arrixaca, 30120 Murcia, Spain; edu_or@yahoo.es

**Keywords:** beta-tricalcium phosphate, hydroxyapatite, mineral trioxide aggregate, vital pulp therapy, scaffolds

## Abstract

Bioceramic materials possess desirable biological properties, highlighting their non-reactivity and osteoconductivity. Their use has been extended in vital pulp treatment. The purpose of this study was to evaluate and compare the effects of beta-tricalcium phosphate (β-TCP), hydroxyapatite (HA), and collagen (C) scaffold with mineral trioxide aggregate (MTA) on the vital pulp of rat molars. Thirty-two molars of Sprague–Dawley rats underwent direct pulp capping with β-TCP/HA/C (n = 16) and MTA (n = 16). After 30 days, the following parameters were evaluated in the tested samples: the degree of pulp inflammation and pulp vitality, the presence of reparative dentin, the homogeneity of the odontoblastic layer, and the presence of pulp fibrosis. No statistically significant differences were observed between HA/β-TCP/C and MTA in terms of the degree of inflammation (*p* = 0.124). Significant differences were found in reparative dentin formation between the treatment groups (*p* = 0.0005). Dentin bridge formation was observed in the MTA-treated group. The local action of HA/β-TCP/C is similar to that of MTA when used as an agent for pulp vital treatment in terms of absence of inflammation and maintenance of pulp vitality, although there are significant differences between both materials regarding the formation of dentin bridges.

## 1. Introduction

Hydroxyapatite (HA) and tricalcium phosphate (TCP) are categorized as bioceramic materials with excellent biological properties, among which their non-reactivity and osteoconductivity can be highlighted [1]. Their use as bone substitutes has been extended in several medical and dental specialties [2,3,4].

TCP is a porous bioceramic material which is resorbable and biocompatible. This material gradually degrades and becomes replaced by bone tissue. In this way, it acts as a scaffold for bone growth [5]. Among the two types of TCP used in bone grafts (alpha and beta), the beta type (β-TCP) presents a greater stability, as exhibited by functional calculations of bone density [6].

TCP and HA have been applied in several dental and maxillofacial procedures, including endodontic treatments [7,8]. The calcium ions released by TCP and its biocompatibility can aid in the stimulation of odontoblasts, thus promoting the formation of reparative dentin. For this reason, it has been used in vital pulp treatment (VPT) as a pulp capping agent [9,10,11]. HA/β-TCP is a biphasic calcium phosphate ceramic (BCP); introduced as a suitable scaffold material which is more effective than pure HA or β-TCP alone [12,13]. BCP has been presented in different formats with varying HA and β-TCP ratios. It has also been combined with stem cells of various origins for bone tissue engineering purposes [14].

Collagen (C) is a natural, bioabsorbable structural protein with which cells can interact and adhere. Collagen sponges and foams have been used as hemostatic agents, scaffolds for tissue repair, and for cell growth support [15]. Likewise, collagen membranes have also been used together with BCP in pathologic extraction sockets with dehiscence defect in dogs’ mandibles [13].

Previous reports have confirmed that the incorporation of a 3D structure, β-TCP and collagen hybrid constructs favor the adhesion and proliferation of human dental pulp stem cells, thus promoting osteogenic/odontoblastic differentiation [16,17]. Similarly, immortalized human dental pulp cells exhibited odontogenic differentiation ability and secretion of dentin sialophosphoprotein (DSPP) when combined with a beta-tricalcium phosphate scaffold and bone morphogenetic protein-2 (BMP2) in an in vivo study [18].

From a biological perspective, it has been shown that some biomaterials like calcium silicate or β-TCPcan directly modify the osteoblastic proliferation rate and differentiation, such as the synthesis of alkaline phosphatase [19,20,21]. It is known that some molecules are directly involved in osteoblast cell differentiation, such as core-binding factor 1 (Cbfa 1), a transcription factor that is necessary for the activation of this process and that regulates the genes responsible for the synthesis of bone-specific proteins [20].

Among VPT procedures, direct pulp capping is indicated when the pulp is visibly exposed (vital pulp exposure) due to caries, trauma, or iatrogenic stimuli such as accidental exposure during tooth preparation or caries removal [22]. Mineral trioxide aggregate (MTA) is a material that has shown high success rates as a pulp capping agent for VPT, both at a clinical, radiological, and histological level. It is widely considered as the gold standard or reference material with which to compare new biomaterials for this type of treatment [23,24]. It has been stated that MTA induces the osteogenic activity of alkaline phosphatase, osteonectin, osteocalcin, and osteopontin, resulting in the formation of a rigid tissue bridge [25]. Some authors have suggested that dentin bridge formation may be stimulated through a protein solubilization process by dentin exposure to MTA, with a consequent modulation of pre-osteoblastic cell gene expression [26]. MTA results in predictable treatments from both a histological and clinical perspective, although it cannot be defined as the ideal material [27]. In fact, some authors report that there is no evidence that this material is better than others to perform vital pulp therapy [28].

A series of compounds with β-TCP have been tested in vitro on pulp cells to verify their biocompatibility and possible use as pulp capping materials [29]. Additionally, in vitro studies regarding BCP have concluded that it can act as an adequate scaffold material for odontogenic differentiation [30,31].

HA/β-TCP’s mechanism of action on osteoblasts in bone may be extrapolated to its action on teeth and odontoblasts. The purpose of the present study was to evaluate the response of vital pulp tissue in rat molars to HA/β-TCP/C when used as a capping agent and compare its histological effects to those of MTA. The null hypothesis is that there are no differences between HA/β-TCP/C and MTA in vivo.

## 2. Materials and Methods

### 2.1. Animals and Surgical Procedure

The Research Ethics Commission of the University of Murcia (Murcia, Spain) approved this study (ID: 292/2017). Eight male Sprague–Dawley rats were used following Royal Decree 1201/2005, law 32/2007, European Directive 2010/63/UE on the protection of animals used for scientific purposes. The average weight of the rats was 230 g approximately, and their average age was 3 months. Rats were kept in cages with appropriate measures for adequate animal welfare and were fed with specific food for experimental animals (Harlan TekladBrand, Global Diets. Code 2014) with an ideal composition, and with periodic toxicity and sanitary analyzes. The animals ate and drank ad libitum. Periodic health checks were carried out on the animals.

Four pulp exposures were performed per rat in healthy first and second maxillary molars. Intramuscular injections with a mixture (at 50%) of 2% xylazine hydrochloride (Rompun^®^. Bayer. Kiel, Germany) and ketamine chlorhydrate 100 mg + clorobutanol 5 mg (Imalgene^®^ 1000. Merial, Barcelona, Spain) were used to anesthetize the rats (dose = 0.2 mL/100 g weight). 0.12% chlorhexidine (PerioAid^®^, Dentaid, Barcelona, Spain) was used to clean the molars’ surface. To access the cameral pulp, a tungsten carbide burr with a 0.8 mm-diameter pyriform shape was used (Dentsply Sirona, York, PA, USA), driven by a turbine with aqueous cooling (KaVo E680L Experttorque, Wien, Austria). Hemostasis was echieved by pressure with sterile cotton balls for a maximum of 5 min. The experimental materials were placed on the pulpusing a small ball applicator. A small amount of test material was applied onto the exposure (approximately 400 ng), enough so that the exposure was sealed entirely by de material. Then, the excess moisture was removed with a dry cotton pellet. Subsequently, it was covered with a thin base of zinc oxide-eugenol (IRM^®^Dentsplay Sirona, York, PA, USA) and the cavity was restored with silver amalgam (Tytin^®^ 29945, Kerr, Scafati, Italy) [32].

### 2.2. Experimental Groups

-Group 1: MTA (n = 16 molars). Pro-Root MTA^®^ (DentsplayMaillefer, Ballaigues, Switzerland) was used as a pulp capping material and was prepared by following its manufacturer’s instructions.

-Group 2: HA/β-TCP/C (n = 16 molars). The mixture was composed of 40% Hydroxyapatite (Sigma-Aldrich, Darmstadt, Germany), 30% β-TCP (Sigma-Aldrich, 49963, Darmstadt, Germany), and 30% collagen (Sigma-Aldrich, C4387 Darmstadt, Germany). All components were powder materials. A creamy consistency was achieved when mixed with distilled water. The particle size was 0.7 mm, and the pore size was 300 µm.

To calculate the sample size (n = 16 per group), a sample size and power calculator was used [33]. An α-risk = 0.05 and β-risk = 0.2 was accepted on a two-tailed test, to find a statistically significant proportion difference which was expected to be 100% in MTA group and 65% in HA/β-TCP/C group. A dropout rate of 10% was assumed.

The material was left to act over a period of 30 days. After this period, rats were sacrificed by a lethal injection of the two anesthetics mentioned above (Rompun^®^ and Imalgene^®^ 1000), and the fragments of maxillae containing the test molars were separated.

### 2.3. Microscope Observation

The maxillary fragments were washed, and organic debris was removed. The tissues were subsequently fixed by immersing the samples in 10% formaldehyde. Then samples were stored for 30 days in 26% formic acid +8.5% sodium citrate (TBD-2, Thermo Shandon, Waltham, MA, USA) to decalcify the hard tissues. Afterwards, specimens were dehydrated in graded alcohol, included in paraffin, and sectioned in 8 µm slices. Every five sections (40 μm) were stained with hematoxylin-eosin and Masson’s trichrome before optical microscope observation (Leica DM 5000 B, Leica Microsystems, Wetzlar, Germany). 4 levels were analyzed per specimen.

### 2.4. Histological Evaluation

According to the histological evaluation criteria of Horsted et al. [34], later modified by Fuks et al. [35], the parameters shown in Table 1 were analyzed.

### 2.5. Statistical Analysis

A descriptive statistical analysis of the histological data was carried out, finding the frequency distribution for each variable. Group data were compared by contingency table analysis using Pearson’s chi-square test and residual analysis to determine significant associations. *p*-values < 0.05 were considered significant.

## 3. Results

### Histological Evaluation

**Figure 1:**
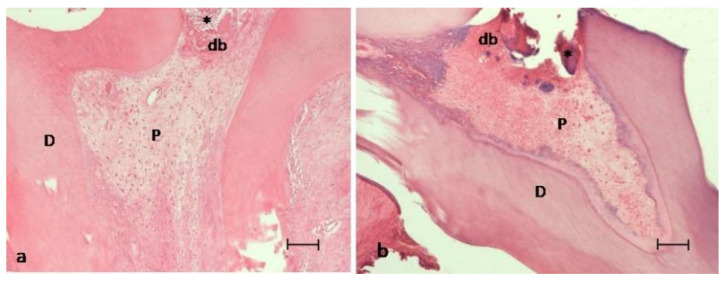
Images of the pulp exposure site of two experimental groups at thirty days post-treatment. (**a**) MTA group. (**b**) HA/β-TCP/C group. Both groups showed a well-developed dentinal bridge (db) formed in contact with the material (asterisk) used for pulp capping. P = viable pulp without necrosis; D = dentin. Hematoxylin and eosin staining. Scale bar: 100 micrometers.

**Figure 2:**
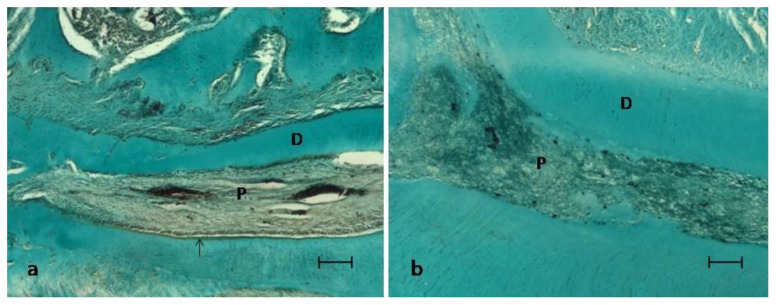
Images of a root canal at thirty days post-treatment. P = viable pulp without necrosis; D = dentin. (**a**) MTA group showed an intact odontoblastic layer (arrow) (**b**) HA/β-TCP/C showed fibrosis along the root canal. Masson’s trichrome staining. Scale bar: 100 micrometers.

In the MTA group, 16 teeth were evaluated. At thirty days, the histological analysis revealed a pulp tissue with the presence of fibrosis (100%) and a tubular reparative dentin in contact with MTA (87.5%) (Figure 1a). A regular odontoblastic layer could be observed, and inflammation was absent in all samples’ dental pulp (Figure 1a and Figure 2a) (Table 2).

In the experimental group treated with HA/β-TCP/C, 16 teeth were also evaluated. Thirty days after treatment, the most common pattern was of a pulp with the presence of blood vessels, without inflammation or necrosis, and with a regular odontoblastic layer in 56.25% of the samples (Figure 2a); being absent in the 43.75% remaining samples (Figure 2b). Fibrosis could be observed in 75% of the samples along the root canal (Figure 2b), and reparative dentin could only be observed in 25% of the samples (Figure 2a) (Table 2).

No statistically significant differences were observed concerning the degree of inflammation or necrosis between the two groups (*p* = 0.124). MTA group exhibited superior results in terms of reparative dentin and dentin bridge formation, with statistically significant differences. (*p* = 0.0005). Lastly, significant differences were identified for the odontoblastic layer (*p* = 0.003) and fibrosis (*p* = 0.03). All samples in the MTA group showed an intact odontoblastic layer and fibrosis (Table 2).

## 4. Discussion

In the present study, rat molars were used to compare the pulps’ response to the direct capping with MTA and HA/β-TCP/C scaffolds. Rat molars and their pulp could act as miniature human molars, since they are very similar at an anatomical, histological, and physiological level. In fact, some authors have stated that human pulp healing is histologically comparable to that of the rat’s pulp [36].

Biomaterials used for VPT procedures should be innocuous for the pulp tissue and its surrounding structures. In the present study, MTA and HA/β-TCP/C groups showed an absence of inflammation and necrosis (100%). The sequence of reparative processes resulting from the application of the tested pulp capping materials after the pulp chamber’s perforation were observed. In brief, the following sequence of pulp repair processes occur: In summary, according to the literature, during the first 15 days, a physiological inflammatory process occurs, which disappears in the following days to give rise to fibrosis [37]. In the present study, at 30 days, significant fibrosis (newly produced collagen) of the pulp was observed adjacent to the perforation, accompanied by a calcification process in the collagen matrix (Figure 1a and Figure 2a). Specifically, fibrosis was found in all specimens in the MTA group, and in 75% of the HA/β-TCP/C group specimens (*p* = 0.03) (Figure 2b). These results confirm that β-TCP is biocompatible. It does not cause any pulp inflammation at 30 days [38]. This results are in accordance with another study in primary molars from pigs, which showed positive results after pulp capping with β-TCP [10].

MTA releases calcium ions when it comes into contact with pulp cells, leading to the expression of osteonectin, bone morphogenetic protein, and osteopontin. MTA solubilizes growth factors such as TGF-b in order to form new tissues [39], and vascular endothelial growth factor (VEGF), which is involved in the process of angiogenesis. Both are crucial in the dentin-pulpal reparative process [40]. Clinically, MTA can stimulate reparative dentinogenesis using a combination of various mechanisms [41]. After applying MTA, localized necrosis of adjacent tissues occurs, which results in the secretion of extracellular matrix by odontoblast-like cells [26] and the subsequent formation of reparative dentin [42].

Regarding β-TCP, evidence describes that it can increase the adhesion and proliferation of human dental pulp stem cells and promote their osteogenic differentiation when used as a 3D-printed biomaterial [16]. Atalayin et al., tested the performance of different scaffolds in vivo for dental pulp stem cells induced for odontogenic differentiation. In their study, BCP scaffolds were used as a reference [43]. Endo et al. studied the implantation of constructs of dental pulp cells and BCP scaffolds into exposed pulp tissue in porcine teeth, resulting in the stimulation of the formation of calcified dentin-like structures [44]. Gu et al., in a recent study on a biphasic calcium phosphate cement modified with β-TCP, showed that this cement exhibits a significant anti-inflammatory effect and a great capacity to regenerate dentin, justifying its potential use in dental tissue engineering [45]. Altogether, evidence points to the potential use of β-TCP in VPT to stimulate dentinal bridge formation.

In fact, other authors affirm that β-TCP promotes dentin formation after pulp exposure in pig teeth. This process may be related to the release of calcium ions after the degradation of β-TCP in contact with pulp cells [10]. However, in the present study, the histological images revealed reparative dentin formation in 25% of the cases in the HA/β-TCP/C group (Figure 1b). These results can be explained by the fact that human mesenchymal stem cells have been shown to proliferate to a greater extent on small granular BCP, like the particles used in this study (300 µm), while large granular BCP promote cell differentiation in vitro [14]. Lobo et al. compared the adhesion, proliferation, viability, and osteogenic potential of these cells on granular BCP with equal HA/β-TCP ratio of diverse particle sizes, and they demonstrated that these biphasic ceramics modulate the in vitro behavior of stem cells depending on their chemical composition and physical characteristics [14]. Higashi and Okamoto compared the effect of two different particle sizes of HA and β-TCP separately (0.04 and 0.3 mm) as capping agents on hard tissue barrier formation after experimental pulpotomy in the dental pulp. Using a small particle size resulted in inflammation, abscess formation, and poor dentin formation [46].

The effects of scaffold porosities on cell viability and differentiation of human dental pulp cells for dentin tissue regeneration were studied by Qader et al. [47]. These authors highlight the impact of different scaffold porosities on the cell microenvironment and demonstrate that BCP scaffolds of 65% porosity can allow human dental pulp cell differentiation for dentin tissue regeneration. In the present study, porosity was not quantified, acting as a potential limitation.

Regarding the results of this study, a healthy and structured pulp was observed with a regular odontoblastic layer both in molars treated with MTA (Group 1, 100%; Figure 1a and Figure 2a) and in those treated with HA/β-TCP/C (Group 2, 56.25%; Figure 1b). Since pulp exposures were not associated with carious pathology, and a suitable coronal sealing was performed, the implication of bacteria as a cause of failure can be practically excluded. Therefore, as shown by the maintenance of the pulp vitality and the absence of inflammation, the biocompatibility of the studied materials can be confirmed [48].

This study’s main limitation was the lack of evidence regarding HA/β-TCP/C-based scaffolds and their potential for pulp-capping treatment. Further investigations on histological and cellular components are recommended to assess the HA/β-TCP/C-based scaffolds. A longer follow-up period is also needed to evaluate the dentin reparation/neoformation.

## 5. Conclusions

HA/β-TCP/C could be potentially used as an agent for vital pulp therapy. The local action of HA/β-TCP/C is similar to that of MTA when used as pulp capping agent, in terms of the absence of inflammation and maintenance of pulp vitality. However, there are significant differences between both materials in terms of dentin bridge formation, favoring MTA. Further clinical studies are needed to confirm the results shown by animal studies.

## Figures and Tables

**Table 1:** Scores attributed for the levels of the histological criteria evaluated.

Pulp Inflammation	0	Absent of inflammation
	1	Mild inflammation
	2	Moderate inflammation
	3	Severe inflammation
	4	Abscess
Pulp Necrosis	0	Absence
	1	Presence
Dentinal bridge and reparative dentin formation	0	Presence
	1	Absence
Odontoblastic layer	0	Regular
	1	Irregular
	2	Absence
Fibrotic tissue	0	Absence
	1	Presence

**Table 2:** Histological results of each experimental group. * *p* < 0.05.

Criteria	Degree	MTA	HA/β-TCP/C	*p* Value
Pulp Inflammation	0	100%	100%	*p* = 0.124
	1			
	2			
	3			
	4			
Pulp Necrosis	0	100%	100%	*p* = 0.124
	1			
Dentinal bridge and reparative dentin formation	0	87.5%	25%	*p* = 0.0005 *
	1	12.5%	75%	
Odontoblastic layer	0	100%	56.25%	*p* = 0.003 *
	1			
	2		43.75%	
Fibrotic tissue	0		25%	*p* = 0.03 *
	1	100%	75%	

## Data Availability

Contact corresponding author for data available.

## Abbreviations

β-TCP	Beta-tricalcium phosphate
HA	Hydroxyapatite
C	Collagen
MTA	Mineral Trioxide Aggregate
HA/β-TCP/C	triphasic calcium phosphate ceramic with Hidoxiapatite, beta-tricalciumphosphate and Collagen
TCP	Tricalcium Phosphate
BCP	Biphasic calcium phosphate ceramic
HA/β-TCP	biphasic calcium phosphate ceramic with Hidoxiapatite and beta-tricalciumphosphate
DSPP	dentin sialophosphoprotein
db	dentinal bridge
P	viable pulp without necrosis
D	dentin
VEGF	vascular endothelial growth factor
VPT	vital pulp treatment
